# An environmental DNA metabarcoding survey reveals generic-level occurrence of scleractinian corals at reef slopes of Okinawa Island

**DOI:** 10.1098/rspb.2023.0026

**Published:** 2023-03-29

**Authors:** Koki Nishitsuji, Tomofumi Nagata, Haruhi Narisoko, Megumi Kanai, Kanako Hisata, Chuya Shinzato, Noriyuki Satoh

**Affiliations:** ^1^ Marine Genomics Unit, Okinawa Institute of Science and Technology Graduate University, Onna, Okinawa 904-0495, Japan; ^2^ Incorporated Foundation Okinawa Environment Science Center, Urasoe, Okinawa 901-2111, Japan; ^3^ Atmosphere and Ocean Research Institute, The University of Tokyo, Kashiwa, Chiba 277-8564, Japan

**Keywords:** coral reef survey, environmental DNA barcoding method, direct diving observation, Okinawa Island

## Abstract

Coral reefs have the highest biodiversity of all marine ecosystems in tropical and subtropical oceans. However, scleractinian corals, keystone organisms of reef productivity, are facing a crisis due to climate change and anthropogenic activities. A broad survey of reef-building corals is essential for worldwide reef preservation. To this end, direct observations made by coral-specialist divers might be supported by another robust method. We improved a recently devised environmental DNA (eDNA) metabarcoding method to identify more than 43 scleractinian genera by sampling 2 l of surface seawater above reefs. Together with direct observations by divers, we assessed the utility of eDNA at 63 locations spanning approximately 250 km near Okinawa Island. Slopes of these islands are populated by diverse coral genera, whereas shallow ‘moats’ sustain fewer and less varied coral taxa. Major genera recorded by divers included *Acropora*, *Pocillopora*, *Porites* and *Montipora,* the presence of which was confirmed by eDNA analyses. In addition, eDNA identified more genera than direct observations and documented the presence of previously unrecorded species. This scleractinian coral-specific eDNA method promises to be a powerful tool to survey coral reefs broadly, deeply and robustly.

## 1. Introduction

Coral reefs in tropical and subtropical waters foster about 30% of all marine life, thus providing one of the most diverse ecosystems on Earth [1]. Reef-building or scleractinian corals (Anthozoa, Cnidaria) create robust reef structure by depositing massive calcium carbonate skeletons. Approximately 1300 scleractinian species, comprising 236 genera and 25 families, have been reported (Integrated Taxonomic Information System or ITIS; https://itis.gov). In the last two and a half decades, however, coral reefs have confronted growing crises due to global climate change and anthropogenic insults. More frequent coral bleaching caused by global warming of seawater, severe storms and predation by crown-of-thorns starfish have degraded and diminished healthy reef ecosystems [2–6]. Since coral reef environments are constantly and dramatically changing, frequent, high-density coral monitoring is essential to assess these changes, in the hope of more effective coral reef preservation.

Conventional coral monitoring depends on direct observation by coral-specialist divers. Such monitoring has greatly contributed to our understanding of corals at various reefs in the Indo-Pacific Ocean, detailing species variation, coverage, health, bleaching, diseases, etc. On the other hand, this method has limited utility for broader surveys. First, it requires coral biologists who are experienced at identifying coral species and are competent divers. In addition, this work is challenging because corals sometimes vary their morphology so drastically as to be unrecognizable [7]. Second, dive time is limited; thus, observations underwater are similarly restricted.

Our previous study demonstrated simultaneous detection of *Acropora* corals and their symbiotic algae from environmental DNA (eDNA) in seawater [8]. Recently, an eDNA metabarcoding method was developed to enable extensive biodiversity monitoring [9–11] including shallow reef corals [12–14]. eDNA barcoding relies on primers that amplify specific regions of mitochondrial and/or nuclear genomes, since sequence differences enable identification of coral genera and species. Early coral eDNA studies used general primers that amplified not only corals, but also other marine invertebrates [12–14]. Therefore, our second study developed other sets of eDNA amplification primers, more broadly applicable, yet specific to scleractinian corals [15]. We developed a set of primers for 12S-ribosomal-RNA genes (12S) to amplify eDNA by which all 36 genera with reported mitochondrial genome sequences (see https://www.ncbi.nlm.nih.gov/genome/browse#!/organelles/, accessed 1 May 2022) can theoretically be identified by sequence differences [15]. Sampling of only 1 l surface seawater over selected reefs successfully identified coral genera present [15].

**Figure 1.**
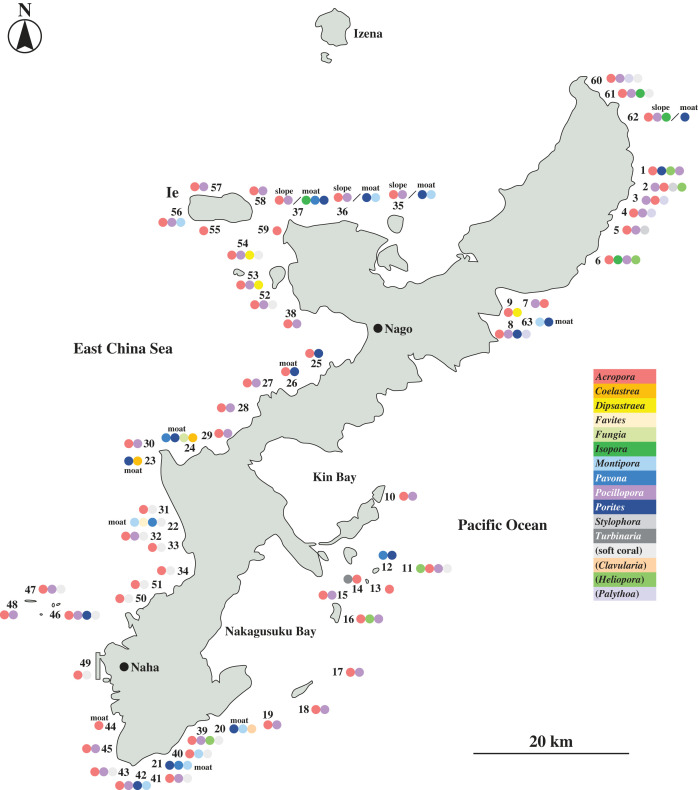
Sixty-three coral reef monitoring locations around Okinawa Island. Collections sites and representative coral genera are shown. Colour codes for genera are shown in the right corner. The order (from left to right) does not always indicate relative dominance of genera. Moats are indicated. Other locations are slopes. At locations #35, 36, 37 and 62, both slopes and moats were examined by diving, whereas only eDNA data were obtained from moats.

This study sought to answer several questions regarding coral-specific eDNA barcoding techniques and interpretation of results. First, since eDNA is a comparatively new technique, its validity and efficiency should be evaluated by simultaneous examination using other methods, such as direct monitoring by coral specialists. If the two sets of results differ, the eDNA method should be used with caution. Second, in the Pacific Ocean, corals in shallow reefs suffered greatly during the 1998 El Niño followed by severe bleaching in 2016–2017, especially *Acropora* [3,16–18]. In inner reef-edge, shallow moat habitats of the Okinawa Island coast, corals suffered severe damage and their recovery has been very slow, although deeper reef slopes appeared less degraded [3,6,16]. Accordingly, in this study we sought to assess the recovery in moats, and to determine which genera are returning. Third, Okinawa Island is located at approximately 26.270–26.739°N latitude and 127.698–128.266°E longitude. Okinawa's east coast faces the Pacific Ocean while its west coast faces the South China Sea (figure 1), with complex seawater currents in various directions. We attempted to determine whether there are differences in coral genera between sites. With these objectives, we examined the utility of recently developed coral eDNA barcoding method.

## 2. Material and methods

### (a) Sampling

Reef monitoring was conducted at 63 coral reef locations in the latter half of 2021 by simultaneous diving observations of corals and collection of surface seawater above these reefs. Names of monitored locations, survey dates, geomorphic classification, latitude, longitude, types of coral community and major coral genera observed are summarized in figure 1, and electronic supplementary material, table S1 and figure S1. Most locations were reef slopes, 3–10 m in depth, and some were inner reef edge moats, 1–3 m in depth (figure 1; electronic supplementary material, table S1).

Two experienced coral reef specialists snorkeled around reefs and recorded coral community types and dominant coral genera (species), according to the conventional Monitoring Sites 1000 Project conducted annually by the Ministry of the Environment of Japan (https://www.biodic.go.jp/moni1000/manual/spot-check_ver5.pdf). Types of coral community included ‘multi-species mixed’, ‘specific species’ and ‘not determined’, where the first and second categories were distinguished by dominant species and their relative abundances (electronic supplementary material, table S1 and figure S1). Dominant species included tabular *Acropora*, branching *Pocillopora* and massive *Porites.* Monitoring of each site required about 30 min with repeated snorkeling. Each diver recorded coral genera based on his or her taxonomic experience. At the end of each day, the divers discussed their observations to reach a consensus regarding major coral genera observed at each site (electronic supplementary material, table S1). Coral coverage of individual reefs was beyond the scope of the present study and was not assessed.

At the same time that divers were observing corals at a given coral reef slope, 3 × 2 l of surface seawater were collected by eDNA researchers in a boat. At moats, the same number of samples was collected by throwing a bucket into the sea. To avoid contamination of seawater samples, containers and bucket were washed with fresh water prior to every sampling. Two-litre samples were filtered individually through 0.45 μm Sterivex filters (Merck) within 5 min after seawater collection. Then 1 ml of RNAlater (Qiagen) was added to the filtrate to prevent DNA degradation. Filters were maintained at 4°C before transfer to a −20°C freezer in the laboratory. Due to heavy waves, we were unable to collect a seawater eDNA sample at site #42.

### (b) eDNA extraction, PCR amplicons and sequencing

eDNA in Sterivex filters was extracted following the Environmental DNA Sampling and Experiment Manual v. 2.1 [19]. Extracted eDNA samples were PCR-amplified using primers, Scle_12S_Fw (5′ -CCAGCMGACGCGGTRANACTTA-3′) and Scle_12S_Rv (5′-AAWTTGACGACGGCCATGC-3′), for mitochondrial 12S rRNA genes, as described in Shinzato *et al*. [15]. These primers were designed to identify 36 scleractinian coral genera, including *Acanthastrea, Acropora, Anthemiphyllia, Astreopora, Coscinaraea, Crispatotrochus, Dipsastraea, Echinophyllia, Favites, Fimbriaphyllia, Fungia, Galaxea, Goniastrea, Goniopora, Heliofungia, Herpolitha, Hydnophora, Isopora, Leptoria, Lobophyllia, Montipora, Orbicella, Pachyseris, Pavona, Pectinia, Platygyra, Plesiastrea, Pocillopora, Polycyathus, Polyphyllia, Porites, Psammocora* and *Turbinaria* [20,21]. All genera are common at Okinawa Archipelago (OA) except for *Anthemiphyllia* (https://www.gbif.org/species/2260364)*, Crispatotrochus* (https://www.gbif.org/species/2259048) and *Polycyathus* (https://www.gbif.org/species/2259234), though they are reportedly present at OA [20]. *Orbicella* is an Atlantic Ocean genus, and its closest relatives in the Pacific Ocean are *Astrea* and *Leptastrea*, both of which are common in the OA [20].

Genomic DNA isolated from an *Acropora tenuis* colony was used as a positive PCR control (data not shown). Tks Gflex DNA Polymerase (Takara) was used for PCR amplification. PCR cycling conditions were 1 min at 94°C, followed by 35 cycles of 10 s at 98°C, 15 s at 60°C and 30 s at 68°C, with an extension of 5 min at 68°C in the final cycle. PCR products were extracted and cleaned with a FastGene Gel/PCR Extraction Kit (NIPPON Genetics Co., Ltd.). Amplicon sequencing libraries of cleaned PCR products were prepared using a KAPA Hyper Prep Kit (NIPPON Genetics) without fragmentation. Libraries were multiplexed and 300-bp paired-end reads were sequenced on a MiSeq platform (Illumina) using a MiSeq Reagent kit v. 3 (600 cycles). The number of sequence reads, total base-pair length, and average and maximum length of reads of each sample are shown in electronic supplementary material, table S2.

### (c) Bioinformatic analysis

Analyses were carried out as described by Shinzato *et al*. [15]. Briefly, after removal of low-quality bases (Phred quality score less than 20) and Illumina sequencing adapters [22], the remaining sequences were merged using USEARCH, v. 11.0.667 [23]. Then, de-noised (error-corrected) operational taxonomic units, called ZOTUs (zero-radius operational taxonomic units), were prepared for each sample. ZOTU sequences from all samples were concatenated and clustered using CD-HIT-EST v. 4.6 with 100% nucleotide identity [24]. Clustered, unique ZOTU sequences were used for the database for mapping. Merged sequences from each sample were mapped to the clustered ZOTUs and numbers of mapped sequences for each ZOTU were counted using the USEARCH ‘otutab’ command with 99% percent identity (-id 0.99).

Identification of ZOTUs originating from scleractinians were selected based on criteria described in our previous study [15]. After selecting scleractinian ZOTUs, mapped reads from the same genera were combined. To remove possible errors and contamination, only genera with more than 0.1% of the total number of mapped reads in a given sample were considered. To infer similarities between samples and sampling locations, hierarchical clustering based on the ward D method was performed using percentages of coral genera. Numbers of ZOTUs at sampling locations are shown in electronic supplementary material, table S2. A rough estimation suggested that 1% of ZOTUs was supported by 28.2 ± 12.3 sequence reads.

During direct observations, divers identified *Stylophora* (electronic supplementary material, table S1)*,* which was not included in our previous eDNA study [15], since the mt genome sequence of *Stylophora pistillata* was deposited in the NCBI database only on June 6, 2021. Therefore, we updated the previously reported informatic tools so that *Stylophora* was also included in the present analysis. Simultaneously, an additional 8 genera were also included (*Agaricia, Catalaphyllia, Diploastrea, Micromussa, Oulastrea, Paraechinophyllia, Physogyra* and *Thalamophyllia*)*.* Therefore, this method is probably able to identify 45 genera, and 43 of these were detected in the present study (electronic supplementary material, figure S2).

Numbers of genera detected by direct observation and eDNA metabarcoding method were statistically compared for slopes and moats, respectively, using the Wilcoxon signed-rank test using R v. 4.2.1 [25]. Overlap coefficients between genera detected by direct observation and eDNA method was calculated for each sampling point.

## 2. Results

With a combination of diver observations and eDNA barcoding, we surveyed scleractinian occurrence at 63 coral reef sites around Okinawa Island (figure 1) from early September to late December 2021, although seawater sampling was unsuccessful at one site (electronic supplementary material, table S1).

### (a) Direct observation by diving

Major genera recorded at each location included *Acropora* (tabulate or branching), *Porites* (massive), *Pocillopora* (branching), *Stylophora*, *Isopora, Dipsastraea, Turbinaria, Montipora, Favites* and *Coelastrea* (this is the new generic name of *Goniastrea*; electronic supplementary material, table S1) and *Pavona* (electronic supplementary material, table S1) (figure 1). Although divers observed that *Heliopora* and *Palythoa* were also abundant, the former is a zoantharian genus and the latter is an octocoral genus; thus, they were not detected by this eDNA method. An overview of the results showed that *Acropora* was dominant at many reefs around Okinawa Island (electronic supplementary material, table S1, figure S1; figure 1), and it appeared to be more common at reefs in the South China Sea (electronic supplementary material, table S1, figure S1; figure 1). By contrast, reefs along the northern part of the Island facing the Pacific Ocean (called nPC) exhibited mixed coral genera, including *Pocillopora, Isopora*, and *Acropora* (electronic supplementary material, table S1; figure S1; figure 1).

**Figure 2.**
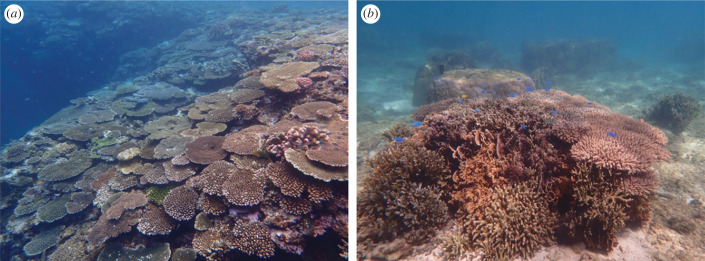
Distribution of various scleractinian corals at a reef slope (#8, Gesashi Uppama East Slope) (*a*) and a moat (#21, Ohdo East Moat) (*b*). Dominant genera in (a) are tabular *Acropora* and branching *Pocillopora* and (*b*) massive and branching *Porites* and tabular *Acropora*.

Of 63 monitoring sites, 51 were slopes (3–10 m in depth; figure 2*a*), 8 were moats (1–3 m in depth; figure 2*b*), and 4 sites were both slopes and moats (#35-37, 62). Direct observations demonstrated clear differences in coral composition between slopes and moats. As mentioned above, *Acropora* was dominant over most slopes except at nPC. By contrast, moats, e.g. sites #20, 21, 22, 23 and 24, fostered a variety of genera including *Porites, Pavona, Montipora* and *Coelastrea* (electronic supplementary material, table S1; figure 1), although fewer *Acropora* were also found in moats (figure 2*b*). This feature was more clearly distinguishable when comparing sites #23 (moat), 24 (moat), 29 (slope) and 30 (slope). These four sites are located along 6 km of the Okinawa coast adjacent to Yomitan and Onna villages. Two slopes (#29 and #30) were dominated by *Acropora*, with *Pocillopora* second (electronic supplementary material, table S1; figure 1). By contrast, moat #23 was covered with *Porites, Coelastrea* and *Pavona,* and #24 with *Pavona, Porites, Fungia* and *Coelastrea,* and a smaller number of *Acropora* (electronic supplementary material, table S1; figure 1).

Further evidence for this tendency came from a comparison of coral genera between moats and adjacent slopes. Sites #35, 36 and 37 constituted such a case (electronic supplementary material, table S1; figure 1). It appeared that slopes were dominated by *Acropora* and *Pocillopora*, whereas the moats were most heavily populated with *Porites, Montipora, Isopora* and *Pavona* (electronic supplementary material, table S1; figure 1). Location #62 exhibited similar composition (electronic supplementary material, table S1; figure 1).

Local differences in coral composition were also observed. For example, sites #25 and #27–29 are along 20 km of the coast of the Onna Village, at popular diving spots. The dominant genus was *Acropora* at all four sites, whereas the next most dominant taxa were *Porites* at site #25 and *Pocillopora* at sites #27–29 (electronic supplementary material, table S1; figure 1). Since the geomorphic structures of these coral reef slopes are similar, this difference is interesting. Another local difference was found at sites #53 and 54, reefs between Ie and Sesoko Islands (figure 1). *Dipsastraea* was dominant at these sites, even though it was not at neighbouring sites (electronic supplementary material, table S1; figure 1).

### (b) eDNA barcoding examination

**Figure 3.**
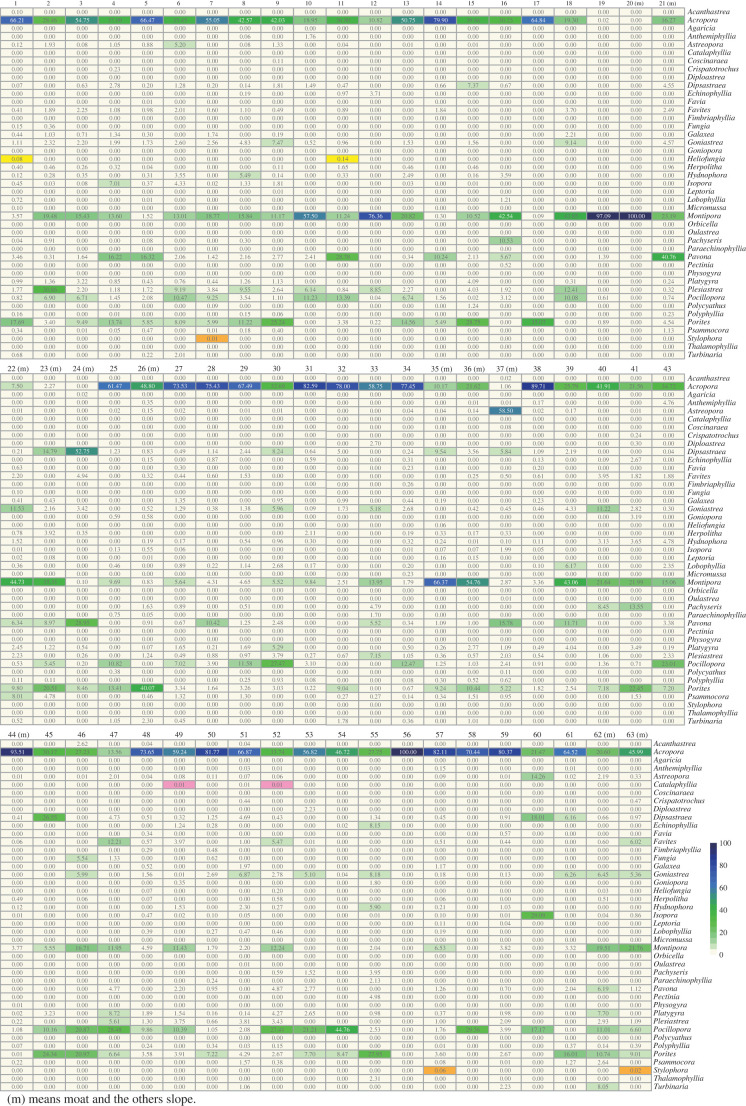
Percentages of ZOTUs mapped to coral genera at each location. Percentages are shown in the heatmap. More detailed data are available in electronic supplementary material, figure S2. Occurrences of *Herpolitha*, *Catalaphyllia* and *Stylophora* are marked with yellow, magenta and brown, respectively.

The eDNA method identified 43 genera, including *Stylophora* (figure 3), but not the octocoral, *Heliopora* (electronic supplementary material, figure S2). The first question regarding the use of eDNA was whether sampling only 2 l of seawater over reefs approximately 3–10 m in depth is sufficient for barcoding analysis. Although our previous study showed that 1 l was enough for amplification of eDNA [15], that survey was limited to one area (near sampling site #27) along a calm, 5 km seashore. The validity of the method over coral reefs with different morphologies and currents was not necessarily assured. However, we obtained amplicons from all samples at 62 sites (no sample was collected at site #42), confirming that 2 l of surface seawater are enough for scleractinian-specific eDNA metabarcoding (figure 3; electronic supplementary material, figure S2).

The second question was whether only one sample is enough to achieve consistent results. As described in the Material and methods section, this study collected triplicate samples of surface seawater from boats, at the same time as diving observations. Triplicate samples from points (#1–9) were sequenced while duplicate samples from the remaining 53 locations (#10–63, except #42) were sequenced (electronic supplementary material, figure S2). We first examined whether triplicate or duplicate samples identified the same coral genera using ZOTU calls. As a result, for 7 of 9 triplicate samples, all three samples proved similar, whereas at the other two sites, two of the three samples were similar, but the third was different. In these cases, we averaged the data from the two similar samples. Among 53 duplicate samples, we judged that the average of the two samples was usable, although results at 5 sites differed slightly (electronic supplementary material, figure S2). Therefore, analysis of at least duplicate samples is recommended.

The third question was a threshold problem. The presence or absence of coral genera was shown by the percent scores of ZOTUs of selected genera per total ZOTU number (figure 3). Scores greater than 5% seem reasonable to use as documentation of the presence of major coral genera (figure 3; table 1; electronic supplementary material figure S2). On the other hand, it is uncertain how to treat samples with scores of 0.5% and below. For example, at location #3, *Acropora, Montipora, Porites* and *Pocillopora* accounted for 54.8%, 15.4%, 9.5% and 6.7% of all ZOTUs, and we concluded that all four genera were present at the reef (figure 3; table 1). In addition, ZOTU percentages were 3.2% for *Platygyra*, 3.0% for *Plesiastrea*, 2.3% for *Favites* and 2.2% for *Caulastrea.* At this site, the average number of reads/ZOTUs was 2265.4 (electronic supplementary material, table S2). This means that 1% ZOTU corresponds to 22.7 Reads. Therefore, we concluded that these four genera were present at location #3.

**Table 1.** Comparison of scleractinian corals called by direct diver obseration and coral-specific eDNA barcoding method.

no	point name	geomorphic classification	direct observation			number of genera detected	eDNA method				number of genera detected	match	overlap coefficient
25	Busenamisaki west	slope	*Acropora*	*Porites*		2	*Acropora*	*Porites*	*Pocillopora*		3	strong	1
27	Onna	slope	*Acropora*	*Pocillopora*		2	*Acropora*	*Pocillopora*	*Montipora*		3	strong	1
28	Onnason Akasaki west	slope	*Acropora*	*Pocillopora*		2	*Acropora*	*Montipora*	*Pocillopora*		3	strong	1
29	Maedamisaki west	slope	*Acropora*	*Pocillopora*		2	*Acropora*	*Pocillopora*	*Montipora*		3	strong	1
30	Zanpamisaki west	slope	*Acropora*	*Pocillopora*		2	*Acropora*	*Pocillopora*	*Dipsastraea*		3	strong	1
31	Toguchi west	slope	*Acropora*			1	*Acropora*	*Montipora*	*Pocillopora*		3	strong	1
32	Mizugama	slope	*Acropora*	*Pocillopora*		2	*Acropora*	*Porites*	*Dipsastraea*		3	moderate	0.5
33	Sunabe	slope	*Acropora*			1	*Acropora*	*Montipora*	*Plesiastrea*		3	strong	1
34	Isa	slope	*Acropora*			1	*Acropora*	*Pocillopora*	*Goniastrea*		3	strong	1
38	Shiokawa Port south	slope	*Acropora*	*Pocillopora*		2	*Acropora*	*Montipora*	*Porites*		3	moderate	0.5
49	Oominezaki Oose	slope	*Acropora*			1	*Acropora*	*Montipora*	*Pocillopora*		3	strong	1
50	Karasuzaki west	slope	*Acropora*			1	*Acropora*	*Porites*	*Goniastrea*		3	strong	1
51	Janase	slope	*Acropora*			1	*Acropora*	*Goniastrea*	*Dipsastraea*		3	strong	1
52	Sesokojima south	slope	*Acropora*	*Pocillopora*		2	*Acropora*	*Pocillopora*	*Montipora*		3	strong	1
53	Minnajima east	slope	*Acropora*	*Pocillopora*	*Dipsastraea*	3	*Acropora*	*Pocillopora*	*Porites*	*Dipsastraea*	4	strong	1
54	Nakanshi east	slope	*Acropora*	*Pocillopora*	*Dipsastraea*	3	*Acropora*	*Pocillopora*	*Porites*		3	moderate	0.66
55	Iejima Funazubaru south	slope	*Acropora*			1	*Porites*	*Acropora*	*Goniastrea*		3	strong	1
56	Iejima West	slope	*Acropora*	*Pocillopora*	*Montipora*	3	*Acropora*				1	strong	1
57	Ieijma Waji north	slope	*Acropora*	*Pocillopora*		2	*Acropora*	*Montipora*	*Pocillopora*		3	strong	1
58	Iejima Isharabaru east	slope	*Acropora*	*Pocillopora*		2	*Acropora*	*Pocillopora*			2	strong	1
59	Aquarium west	slope	*Acropora*			1	*Acropora*	*Pocillopora*	*Montipora*		3	strong	1
45	Kyan Port west	slope	*Acropora*	*Pocillopora*		2	*Acropora*	*Dipsastraea*	*Porites*	*Pocillopora*	4	strong	1
													
1	Adagashima north	slope	*Acropora*	*Porites*		2	*Acropora*	*Porites*	*Montipora*		3	strong	1
2	Adagashima south	slope	*Pocillopora*	*Acropora*	*Stylophora*	3	*Plesiastrea*	*Acropora*	*Montipora*	*Pocillopora*	4	moderate	0.66
3	Ishikinazaki southwest	slope	*Pocillopora*	*Acropora*		2	*Acropora*	*Montipora*	*Porites*	*Pocillopora*	4	strong	1
4	Katsusenozaki south	slope	*Acropora*	*Pocillopora*		2	*Acropora*	*Pavona*	*Porites*	*Pocillopora*	4	strong	1
5	Aha south	slope	*Acropora*	*Pocillopora*	*Stylophora*	3	*Acropora*	*Pavona*	*Porites*	*Dipsastraea*	4	partial	0.33
6	Pumped Storage Hydropower Station southeast	slope	*Isopora*	*Pocillopora*	*Acropora*	3	*Acropora*	*Montipora*	*Pocillopora*		3	moderate	0.66
7	Higashison Miyagi Unse south	slope	*Acropora*	*Pocillopora*		2	*Acropora*	*Montipora*	*Pocillopora*		3	strong	1
8	Gesashi Uppama east	slope	*Acropora*	*Pocillopora*	*Porites*	3	*Acropora*	*Montipora*	*Porites*	*Pocillopora*	4	strong	1
9	Gesashi north	slope	*Acropora*	*Dipsastraea*		2	*Acropora*	*Porites*	*Montipora*		3	moderate	0.5
10	Ikeijima east	slope	*Acropora*	*Pocillopora*		2	*Montipora*	*Acropora*	*Pocillopora*		3	strong	1
11	Ukibaru Yokobishi east	slope	*Acropora*	*Pocillopora*		2	*Acropora*	*Pavona*	*Pocillopora*		3	strong	1
12	Ukibaru Yokobishi south	slope	*Pavona*	*Porites*		2	*Montipora*	*Acropora*	*Palythoa*		3	no match	0
13	Minamiukibaru southeast	slope	*Acropora*			1	*Acropora*	*Montipora*	*Porites*		3	strong	1
14	Minamiukibaru south	slope	*Turbinaria*	*Acropora*		2	*Acropora*	*Pavona*	*Montipora*		3	moderate	0.5
15	Ginogiiwa northeast	slope	*Acropora*	*Pocillopora*		2	*Porites*	*Acropora*	*Montipora*		3	moderate	0.5
16	Tsukenjima Agihama east	slope	*Acropora*	*Pocillopora*		2	*Montipora*	*Acropora*	*Pachyseris*	*Pocillopora*	4	strong	1
17	Uganiwa south	slope	*Acropora*	*Pocillopora*		2	*Acropora*	*Porites*			2	moderate	0.5
18	Kudakajima Erabuiwa east	slope	*Acropora*	*Pocillopora*		2	*Montipora*	*Acropora*	*Pocillopora*		3	strong	1
19	Kumakajima south	slope	*Acropora*	*Pocillopora*		2	*Montipora*				1	no match	0
39	Ohjima south	slope	*Acropora*	*Pocillopora*		2	*Montipora*	*Acropora*	*Porites*		3	moderate	0.5
40	Mabuni south	slope	*Acropora*	*Montipora*		2	*Acropora*	*Montipora*	*Goniastrea*		3	strong	1
41	Ohdo	slope	*Acropora*	*Pocillopora*		2	*Acropora*	*Montipora*	*Porites*		3	moderate	0.5
43	Arasaki west	slope	*Acropora*	*Pocillopora*		2	*Acropora*	*Pocillopora*	*Montipora*		3	strong	1
46	Chiibishi Kamiyama south	slope	*Acropora*	*Pocillopora*	*Porites*	3	*Acropora*	*Pocillopora*	*Porites*		3	strong	1
47	Chiibishi Nagannu north	slope	*Pocillopora*	*Acropora*		2	*Pocillopora*	*Acropora*	*Montipora*		3	strong	1
48	Chiibishi Nagannu west	slope	*Acropora*	*Pocillopora*		2	*Acropora*	*Pocillopora*	*Montipora*		3	strong	1
60	Usahama east	slope	*Acropora*	*Pocillopora*		2	*Isopora*	*Acropora*	*Dipsastraea*	*Pocillopora*	4	strong	1
61	Oku Port north	slope	*Acropora*	*Pocillopora*	*Isopora*	3	*Acropora*	*Porites*	*Goniastrea*		3	partial	0.33
													
26	Afuso north	moat	*Acropora*	*Porites*		2	*Acropora*	*Porites*	*Turbinaria*		3	strong	1
35	Kourijima Tokeihama	moat	*Porites*	*Montipora*		2	*Montipora*	*Acropora*	*Porites*		3	strong	1
36	Nakijinson Nagahama	moat	*Porites*	*Montipora*		2	*Montipora*	*Acropora*	*Porites*		3	strong	1
37	Bisezaki east	moat	*Isopora*	*Pavona*	*Porites*	3	*Astreopora*	*Pavona*	*Porites*		3	moderate	0.66
44	Itoman Port Kurantogai north	moat	*Acropora*			1	*Acropora*	*Montipora*	*Pocillopora*		3	strong	1
20	Ohjima south	moat	*Porites*	*Montipora*		2	*Montipora*				1	strong	1
21	Ohdo east	moat	*Porites*	*Pavona*	*Montipora*	3	*Pavona*	*Montipora*	*Acropora*	*Porites*	4	moderate	0.66
22	Mizugama	moat	*Montipora*	*Favites*	*Pavona*	3	*Montipora*	*Goniastrea*	*Porites*	*Pavona*	4	partial	0.33
23	Zanpamisaki west	moat	*Porites*	*Coelastrea*		2	*Montipora*	*Porites*	*Dipsastraea*	*Goniastrea*	4	moderate	0.5
24	Maedamisaki west	moat	*Pavona*	*Porites*	*Fungia*	3	*Dipsastraea*	*Pavona*	*Porites*		3	partial	0.33
62	Kunigamison Akasaki north	moat	*Porites*			1	*Acropora*	*Montipora*	*Pocillopora*	*Porites*	4	strong	1
63	Gesashi Uppama east	moat	*Montipora*	*Porites*		2	*Acropora*	*Montipora*	*Pocillopora*		3	moderate	0.5

In addition, eDNA likely provided evidence for genera not yet known from Okinawa Island. For example, two species in the genus *Heliofungia* have been reported [3], *H. actiniformis* (Quoy & Gaimard 1833) and *H. fralinae* (Nemenzo 1959), the latter being recorded in southern Asia, including Japan. However, this species reportedly reaches its northern limit at Miyako Island, and there have been no reports of its presence at Okinawa or Amami Islands. This eDNA analysis identified *Heliofungia* at slopes #1 and #11 (figure 3, marked with yellow). Although the scores seemed low (0.08% for #1 and 0.14% for #11), these scores corresponded to 1.3 (1,673.6 × 0.0008 = 1.3) and 4.4 Reads (3,119.6 × 0.0014 = 4.4) (electronic supplementary material, table S2). That is, ZOTU scores suggest a more northward distribution of *H. fralinae,* reaching at least as far as Okinawa Island.

Another example was the genus *Catalaphyllia*, which comprises only one species, *C. jardinei* [26]. *C. jardinei* has been recorded at non-reef areas of Japan, including Miyazaki, Kochi, and recently, Amami Island, at 30 m depth [27], but not at Okinawa Island. This study recorded it at sites #49 and 52 (0.01% score; figure 3, marked with magenta). The score corresponded to Reads count of 1.7 (2,431.4 × 0.0001 = 1.7) at #49 and 4.0 (3978.6 × 0.0001 = 4.0) at #52 (electronic supplementary material, table S2). Therefore, although these suggestions should be examined further, it is likely that this represents a new record of a small, cryptic genus that had not been reported from Okinawa Island previously.

### (c) Match of results from two methods

To evaluate this scleractinian coral-specific eDNA method, we compared results of eDNA barcoding with direct observations (table 1). As mentioned above, *Acropora* was the dominant genus at most spots. To simplify comparisons, in table 1, we first listed slope sites facing the South China Sea, those facing the Pacific Ocean, and then moats (table 1). For comparison of each point, we listed up to three major genera called by divers and the three most abundant genera identified by eDNA (table 1).

Numbers of genera detected by eDNA were significantly larger than direct observation in both slopes (Wilcoxon signed-rank test, *p* < 0.001) and moats (Wilcoxon signed-rank test, *p* < 0.05). We obtained overlap coefficients (1, 0.66, 0.5, 0.33 and 0) between genera detected by direct observation and eDNA method for each sampling point, and we allocated them to four classes based on the overlap coefficient. That is, strong matches included those with an overlap coefficient of 1. Moderate matches had coefficients from 0.5 to 0.66. Partial matches had coefficients of 0.33, and non-matches had coefficients of 0 (table 1). As a result, 41 of 62 points were well matched (67%), 15 were moderately matched (24%), 4 were partially match (6%), and only 2 points showed no match (3%). In other words, at more than 91% (67%+24%) of monitored locations, eDNA results were confirmed by direct observations.

At 25 slope sites (#25–34, #35–38 and # 49–59), genera identified by eDNA method were well matched to those identified by divers. At most points, the dominant genus was *Acropora*, followed by *Pocillopora, Porites* and/or *Montipora*. In general, both methods identified these genera as dominant and subdominant genera, respectively (table 1). For example, as previously mentioned, sites #25 and #27-#29 are along the coastline of Onna Village, and although direct observations identified *Acropora* as the dominant genus at all four sites, the next most abundant genus at #25 was *Porites* while it was *Pocillopora* at sites #27-29 (table 1). eDNA also identified the same genera as secondary genera (figure 3; table 1). Since the two groups of coral reef slopes are similar, it is interesting to ask how this difference originated. This will be examined in future studies. At sites #53 and 54, located at reefs between Ie and Sesoko Islands, *Dipsastraea* was dominant, even though at neighbouring sites, it was not (table 1; figure 1). Both direct observations and eDNA method detected this taxon (table 1).

Interestingly, *Acropora* was more frequently called by direct observation and *Montipora* was more frequently called by eDNA (table 1). This is partially because branching and tabular *Acropora* are easily spotted, while many *Montipora* species are encrusting taxa and not readily observed. Another example of poorly matched assessments was seen at site #37, in a moat, *Isopora* was identified as the dominant genus by direct obversion, and this site has been recognized as an ideal place to see *Isopora.* On the other hand, eDNA method identified *Astreopora* as dominant, and *Isopora* appeared eighth in abundance (figure 3; table 1). Therefore, the two methods did not always yield the same conclusion.

## 4. Discussion

### (a) Coral eDNA barcoding method

Coral eDNA barcoding requires cautious interpretation of results, since the method is based on several assumptions. Corals constantly secrete mucus, which probably provides most of the coral eDNA in seawater. Coral cellular debris may be another source of eDNA. One assumption is that all coral species secrete the same amount of mucus (eDNA) or debris per unit of body surface. If this differs among genera, data may require adjustment. This assumption needs to be examined and such a study is now underway in our laboratory.

The velocity of surface currents and the magnitude of waves are also problematic. If currents are quite strong, eDNA may not correspond just to that secreted by the reef below, but from neighbouring reefs as well. In this study, of some triplicate samples, one replicate identified a different genus than the other two (electronic supplementary material, figure S2). Therefore, single samples at strong current sites seem inadequate, and at least two replicates should be examined. In addition, our eDNA method is specific to scleractinian corals and cannot detect soft corals or other cnidarians. Therefore, if a given spot was covered by many soft corals, a large amount of soft coral eDNA may affect estimates of scleractinian coral eDNA.

In addition, this study showed that results obtained from direct observation are not completely same as those detected by eDNA method. For example, *Acropora* was more frequently called by direct observation and *Montipora* was more frequently called by eDNA method (table 1). Another example of poorly matched assessments was seen at a moat, site #37. *Isopora* was identified as the dominant genus by direct obversion, but eDNA method identified *Astreopora* as dominant, and *Isopora* appeared eighth in abundance (figure 3; table 1). Therefore, the two methods do not always yield the same conclusion. This should be considered when interpreting results.

In other words, constant improvement of the eDNA barcoding method is required. The bioinformatic method of our previous study based on mt sequence information of 36 genera detected 23 genera from eDNA collected at sites near #27 [15]. However, those primers could not detect *Stylophora.* Therefore, this study improved the method and did detect this genus (Material and Methods). Updates of target mt genome sequences may be important for future identification of a greater number of corals by this method. Another key issue of eDNA method is that the present methodology cannot assess coral coverage at a given reef. This problem should be challenged in the future by incorporation of various new techniques and ideas.

### (b) Scleractinian corals at Okinawa Island

Our survey examined coral diversity at 63 coral reef locations around Okinawa Island. Both diving observations and eDNA method clearly showed that *Acropora* is the dominant genus at most slopes around Okinawa, followed by *Pocillopora, Porites* and *Montipora* as sub-dominant genera. This tendency is more conspicuous at reef slopes facing the South China Sea (table 1; figure 1). The diversity of coral genera is higher on slopes facing the Pacific Ocean than the South China Sea (figure 1) and also among neighbouring locations. This may be caused by seashore-specific ecological and/or geomorphic features, although the basis of this difference need to be investigated in future studies.

In addition to chronic anthropogenic impacts such as soil run-off [3,6], coral reefs around Okinawa Island suffered a mass bleaching event in 1998 and 2016–2017 [3,16–18], and outbreaks of crown-of-thorns starfish in the 2000s [5,28], resulting in low coral coverage up to the 2020s, especially at moats. The recovery of coral coverage from such problems has been studied extensively [3,11,16–18], although their evaluation is not always the same. The quality and quantity of coral genera demonstrated by this study at the 63 locations surround the islands appears higher than we expected, suggesting gradual recovery of scleractinian corals, especially at slopes.

The moats we examined in this study are places that are known to have gradual appearance of corals, while most moats have still fewer corals at Okinawa Island than before. This survey demonstrates remarkable differences in the diversity of coral genera in slopes and moats. By contrast to the dominance of *Acropora* and *Pocillopora* on reef slopes, *Porites* and *Montipora* appeared dominant in moats. The former are branching and/or tubular in morphology, while the latter are massive, branching and/or foliose. The reason for this difference may be tolerance to strong waves, fluctuations in water temperature, and turbidity-sedimentation caused by terrestrial input. Since foliose and branching species of *Montipora* are more fragile to waves while branching and massive species of *Porites* and branching *Montipora* are more tolerant to turbidity-sedimentation and high water-temperature. Insolation intensity may also explain these differences. Nevertheless, long-term monitoring of scleractinian corals in slopes and moats is essential to understand the recovery of healthy coral reefs at this island.

### (c) Future perspective

As such, the eDNA method involves intrinsic technical problems. Nevertheless, overall results of eDNA method are comparable with those obtained by diving observations. That is, the two types of results agree at nearly 90% of locations examined in this study (table 1). This provides strong support for eDNA method as a powerful tool to monitor coral reefs more broadly, deeply and robustly than conventional diving surveys. Our previous eDNA study surveyed coral genera in May 2021 at three points along the Onna Village seashore and recorded abundances of *Acropora* (62%), *Pocillopora* (15%), *Montipora* (4%), *Dipsastraea* (2%), *Porites* (2%), *Favites* (2%) and others [15]. Present diving observations at point #27 in October 2021, closest to the previously surveyed area, recognized *Acropora* as dominant and *Pocillopora* as sub-dominant (table 1). In addition, the present eDNA barcoding found occurrences of *Acropora* (73%), *Pocillopora* (7.5%), *Montipora* (5%), *Porites* (3%), *Fungia* (1%), *Platygyra* (1.7%), *Favites* (0.5%) and *Dipsastraea* (0.5%) (figure 3). This provides further support for the robustness of coral-specific eDNA metabarcoding.

In addition, only 1 l of surface seawater was sufficient for eDNA surveying [11–15]. Therefore, occurrence of scleractinian corals can be robustly monitored using eDNA, hopefully enabling more efficacious plans for coral reef preservation and restoration. In the last two and half decades, dominant scleractinian coral taxa have confronted growing crises due to global climate change and anthropogenic insults. It is urgent to document the present status of corals in reefs worldwide, including at isolated islands in the Pacific Ocean. More comprehensive monitoring of such reefs can be accomplished using this eDNA method.

## Data Availability

The dataset used in this study is publicly accessible: https://doi.org/10.5061/dryad.5dv41ns9j [29]. Electronic supplementary material is available from Figshare [30].
